# Vitamin D Status in Children in Greece and Its Relationship with Sunscreen Application

**DOI:** 10.3390/children8020111

**Published:** 2021-02-05

**Authors:** Gavriela Maria Feketea, Ioana Corina Bocsan, Georgios Tsiros, Panagiota Voila, Luminita Aurelia Stanciu, Mihnea Zdrenghea

**Affiliations:** 1 Department of Hematology, “Iuliu Hatieganu” University of Medicine and Pharmacy, 400000 Cluj-Napoca, Romania; Fechetea.Gabriela@umfcluj.ro (G.M.F.); mzdrenghea@umfcluj.ro (M.Z.); 2 Department of Pediatrics, Hospital Unit of Amaliada, General Hospital of Ileia, Amaliada, 27200 Ileia, Greece; 3 Department of Pharmacology, Toxicology and Clinical Pharmacology, Iuliu Haţieganu University of Medicine and Pharmacy, 400337 Cluj-Napoca, Romania; 4 Family Medicine Department, Health Center of Gastouni, 27300 Ileia, Greece; geotsiro@otenet.gr; 5 Clinical Chemistry Department, Private Laboratory Medicine, Amaliada, 27200 Ileia, Greece; zeta-med@otenet.gr; 6 National Heart and Lung Institute, Imperial College London, London W2 1PG, UK; l.stanciu@imperial.ac.uk; 7 Department of Hematology, Ion Chiricuta Oncology Institute, 400010 Cluj-Napoca, Romania

**Keywords:** vitamin D, 25-hydroxyvitamin D, sunshine, vitamin D deficiency, vitamin D insufficiency, vitamin D sufficiency, sunscreens

## Abstract

The aim of this study was to characterize the prevalence and seasonal variation of vitamin D (vit D) deficiency/insufficiency in healthy children and adolescents in Greece, and to explore its relationship with the use of sunscreens. The serum level of 25-hydroxy-vitamin D (25(OH)D) was measured in 376 children and adolescents (184 males and 192 females) with a mean age of 7.6 ± 4.9 years, at different time points over a period of 13 months. The prevalence of low serum 25(OH)D level, including deficiency and insufficiency, was 66.2%. The lowest mean 25(OH)D was observed in the month of January (17.9 ± 6.8 ng/mL) and the highest in September, July, August, and October (34.6 ± 8.7, 33.0 ± 9.4, 30.1 ± 8.2, and 30.1 ± 10.6 ng/mL, respectively). Higher levels of serum 25(OH)D were detected in the children to whom sunscreens had been applied on the beach (*p* = 0.001) or off the beach (*p* < 0.001). The subjects with deficiency and insufficiency were significantly older than those with normal levels of 25(OH)D, but no significant differences were demonstrated according to gender. This study emphasizes the high prevalence of low serum levels of 25(OH)D and their seasonal variation in children living in a region characterized by many hours of sunshine. Our data suggest that the real-life use of sunscreens during the summer months allows sufficient sunlight to be received to enable production of vit D at a level adequate to maintain normal serum levels. Vit D supplements should be given to children during the months of lower sun exposure.

## 1. Introduction

In the Mediterranean and other countries with many hours of sunshine, vitamin D (vit D) deficiency is common, despite the presence of sunlight almost all year round. Although the use of sunscreens is recommended to reduce the negative effects of exposure to the sun, there is a risk of them not being used, because of the view that they may predispose to vit D deficiency.

### 1.1. Vitamin D

All vitamins are essential constituents of a healthy diet [1]; nevertheless, for vit D, the major sources are dietary intake, skin synthesis, and vit D supplementation. Although it is referred to as a “vitamin”, vit D is actually a fat-soluble steroid pre-hormone. It can be produced in the skin from sunlight exposure [2], which is more efficient in vit D production and maintenance of vit D levels in the body than dietary intake. Lagunova and colleagues showed that a twice-weekly whole-body sunbed exposure is equivalent to 2000 IU daily of oral vit D supplementation for 30 days, and this was enough to achieve and maintain serum levels of 25-hydroxy-vitamin D (25(OH)D) in the normal range in more than half of participants [3].

Vit D2 (ergocalciferol) is derived from intake of vegetable foods and vit D2 supplementation, while vit D3 (cholecalciferol) is derived from intake of animal foods, vit D3 supplements, and skin production [4]. Ultraviolet B (UV-B) radiation from sunlight penetrates into the skin and transforms provitamin D3 (7-dehydrocholesterol) into pre-vitamin D3 (pre-cholecalciferol), which is further thermally converted and released into the bloodstream as vit D3 (cholecalciferol) [5]. Ergocalciferol and cholecalciferol are converted in the liver to 25(OH)D2 and 25(OH)D3, respectively, by the enzyme 25–hydroxylase, and then to the most active form of vit D, 1,25 dihydroxyvitamin D (1,25(OH)2D), by the enzyme 1-alpha-hydroxylase in the kidney [6]. In addition, many target cells are able to convert 25(OH)D to 1,25(OH)2D, locally, and the activated form binds to nuclear vit D receptor (VDR). 1,25(OH)2D also exerts non-genomic actions. The non-genomic mechanisms, downstream of nuclear and/or membrane VDRs complexed to caveolin1, include the activation of certain intracellular signaling molecules [7]. Thus, 1,25(OH)2D mediates rapid responses associated with cell proliferation, differentiation, and apoptosis [8].

Apart from the classical functions in calcium–phosphorus homeostasis and control of bone metabolism, vit D plays a critical role in the modulation of immune function [2]. According to current evidence, the differences in sunlight-dependent production of vit D are influenced by season, latitude, time of day, time of exposure, skin pigmentation, and age [9]. Some studies, especially nonclinical studies, suggest that the use of sunscreen is one of the factors leading to low levels of vit D, but there is disagreement about this [10].

Vit D deficiency, insufficiency, and sufficiency are defined as serum levels of 25(OH)D of <20 ng/mL, 20–30 ng/mL, and >30 ng/mL, respectively (<50 nmol/L, 50–75 nmol/L, and >75 nmol/L, respectively) [11].

### 1.2. Ultraviolet (UV) Radiation

Ultraviolet radiation (UV) can be subdivided into UV-A, -B, and -C wave radiation. UV-A has the longest wavelengths (315–400 nm) and the lowest energy, UV-C has the shortest wavelengths (100–280 nm) and highest energy, and UV-B is between the other two. Atmospheric ozone absorbs UV-C; thus, sunlight reaching human skin is predominantly UV-A (90–95%) and UV-B (5–10%) [12]. Additionally, as the solar zenith angle increases, the amount of UV-B radiation reaching the earth’s surface and, thus, skin exposure are reduced. This angle is increased when the sun is not directly overhead, as occurs at higher latitudes in the early morning and late afternoon, and during the winter months [13].

Personal UV exposure depends not only on the intensity of sunlight in a particular location on earth, but also on the time spent outdoors and the use of shade, UV-protective clothing, and sunscreens [12]. UV irradiation in the summer contributes to the development of skin cancer, while low irradiation in the winter contributes to conditions associated with vit D deficiency [14], as the amount of UV-B radiation absorbed by the skin directly influences the vit D status [15].

### 1.3. Sunscreens

A sunscreen is a sun protection compound applied to the skin, which should block or absorb radiation in the UV range 290–400 nm, in order to reduce the dose of UV that reaches the skin’s surface. The UV filters in sunscreens may be physical or chemical agents. Two physical, inorganic agents are used: titanium dioxide, which provides strong UV-B and some UV-A protection, and zinc oxide, which provides good UV-A and UV-B protection. Most of the chemical, organic agents absorb UV-B radiation, but only a few offer UV-A protection [16]. Exposure to strong sunlight can produce sunburn and skin damage, and it may predispose to the development of skin cancer. The amount of UV-B radiation absorbed by the skin influences the vit D status; thus, it is possible that the use of sunscreens, through their UV-B protection, could result in vit D deficiency [17]. Despite this potential risk, the application of sunscreens in childhood is a firm recommendation of several leading scientific organizations, as a part of sun-safe behaviors [18].

The aim of this study is to characterize the prevalence and seasonal variation of vit D deficiency/insufficiency and its relationship with the use of sunscreens in healthy children in a sunny country, in real-life conditions.

## 2. Materials and Methods

We conducted a prospective 13-month study, from September 2018 through September 2019 in Amaliada, Greece. The participants in this study were recruited from the children who were addressed to our laboratory for their routine blood testing.

The study was reviewed and approved by the hospital ethics and scientific committee, and written informed consent was obtained from the parents of all the study children. Healthy white subjects aged 1 to 18 years, both males and females, with a body mass index (BMI) below the 85th percentile for age and sex, were enrolled, throughout the year of the study. All children visited a beach almost every day during the sunshine months as they were residents of the study region. Moreover, they did not have risk factors for development of vit D deficiency. Exclusion criteria were the administration of vit D supplements during the previous year, chronic health conditions, and acute diseases during the 2 previous months. No differences were recorded in religious practices affecting dietary vit D intake and type of clothing. A face-to-face interview was conducted with each parent and child to collect demographic data and details of the habits regarding sun exposure and sunscreen (SPF > 15) use during the previous 2 months. Regarding the sunscreen, they were asked if it was applied before going to the beach or on the beach. We recorded any data regarding the use of other protective measures than sunscreen use because the authors considered that the mothers could answer if the children had a protective clothing, hat, or sunglasses, but they could not exactly specify how long they really used them. Depending on the school they attended and, thus, their daily program, the children were classified into three age groups: infancy and early childhood (1–4 years), middle childhood (5–12 years), and adolescence (13–18 years).

A venous blood sample was collected from each participant during the scheduled visit. Following collection, the samples were immediately spun, frozen, and stored at −22 °C, with no further freeze–thaw cycles until the time of 25(OH)D analysis. 25(OH)D was measured with the electrochemiluminescence binding assay, used on a Cobas e-411 immunoassay analyzer. Calibration and quality control were performed according to manufacturer’s recommendations. The laboratory followed the established procedures for corrective measures when the values fell outside the defined limits.

### Statistical Analysis

Analyses were conducted using SPSS statistical software (version 22.0). Quantitative variables were expressed as mean values and standard deviation (SD). Qualitative variables were expressed as absolute and relative frequencies. An independent-sample Student’s *t*-test was used for the comparison of mean values between study groups. For the comparison of proportions, chi-square and Fisher’s exact tests were used. Analysis of variance (ANOVA) was performed for the comparison of mean values across three groups. Pearson correlation coefficient was used to explore the association between two continuous variables. All reported *p*-values are two-tailed. Statistical significance was set at *p* < 0.05.

## 3. Results

The study group consisted of 376 children and adolescents (184 males and 192 females) with a mean age of 7.6 ± 4.9 years. The mean serum level of 25(OH)D was 26.7 ± 10.4 ng/mL. Vit D deficiency as defined above was recorded in 27.1% of the children and insufficiency was recorded in 39.1%. The values of vit D according to demographic data, season, and use of sunscreen are presented in Table 1.

The prevalence of low 25(OH)D concentration, including deficiency and insufficiency, in infants, children, and adolescents was 55.2%, 71.2%, and 75% respectively, with no gender difference. A greater age was found to be associated with lower levels of 25(OH)D.

**Figure 1:**
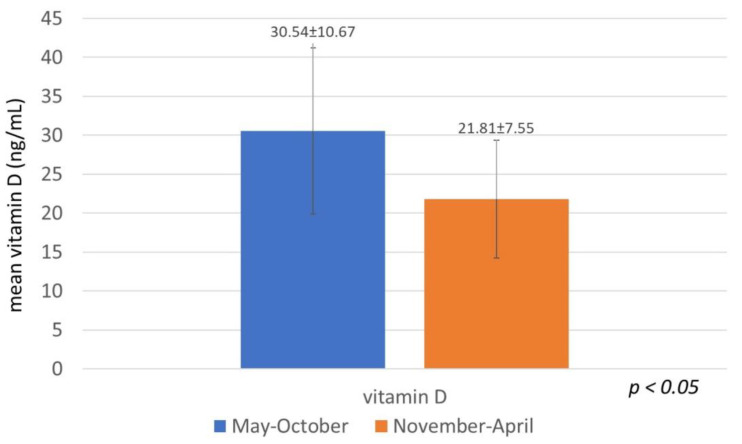
Mean serum level of vitamin D (25(OH)D, ng/mL) in healthy children in Greece during the months May–October and November–April.

The infants (1–4 years) had a higher mean level of 25(OH)D than both the 5–12 year age group and the adolescents (13–18 years), specifically, 29.9 ng/mL vs. 25.67 ng/mL and 23.21 ng/mL, respectively, but the differences were not statistically significant. No gender differences were observed in the mean level of 25(OH)D even after division into age groups. A significant seasonal and monthly variation in the mean level of 25(OH)D was found, with lower levels recorded in the months November to April than in the months May to October (Figure 1).

A comparison of the proportion of children and adolescents with vit D deficiency and insufficiency (Table 2) showed that children with deficiency or insufficiency were significantly older than those with normal serum levels of 25(OH)D.

**Figure 2:**
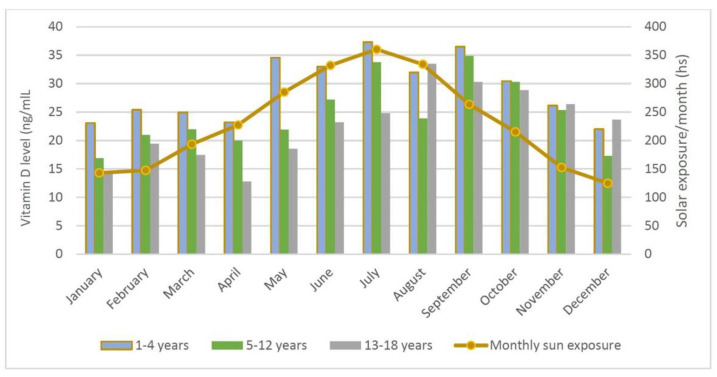
Mean serum concentration of 25(OH)D (ng/mL) in healthy children in Greece (*n* = 376), according to month and age, depending on the mean monthly hours of sunshine.

Adolescents (13–18 years) were deficient/insufficient in vit D during June and July, in contrast to the infants (Figure 2).

Overall, a lower proportion of children with vit D deficiency was found in the summer and autumn months. Regarding the use of sunscreens, significantly higher serum levels of 25(OH)D were recorded in the children that used sunscreens on and off the beach (33.8 ng/mL vs. 24.9 ng/mL) or only on the beach (32.5 ng/mL vs. 23.7 ng/mL) than in those that did not use sunscreen.

## 4. Discussion

The present study showed that vit D deficiency/insufficiency is a frequent disorder even in sunny regions and it has seasonal and age-related variations. The use of sunscreens did not reduce the status of vit D.

The authors excluded, from this study, infants under 1 year of age, as, in Greece, this group is characterized by a very low rate of vit D deficiency, due to the policy of fortification of infant formula and the recommendation for vit D supplementation of breastfeeding infants. Additionally, in children younger than 6 months, it is recommended to avoid using sunscreen and to limit sun exposure. Empirically, sun exposure without sunscreen is extremely limited in the age group of 6–12 months. None of the children selected for this study received vit D supplements in the previous 12 months, in order to avoid the possible influence of supplement on their serum levels of 25(OH)D.

In order to reduce the bias, the authors also excluded from the analysis children with BMO-for-age above the 85th percentile [19], because of the well-established inverse relationship between vitamin D status and obesity [20,21]. Autier and colleagues concluded from two consecutive reviews that a low serum level of 25(OH)D is a marker of ill health, and that inflammatory processes involved in different diseases would reduce the 25(OH)D level [22,23]. On the basis of these findings, we excluded from our study children with chronic diseases, and those who had an acute illness during the previous 2 months.

The children in this study lived in the county of Ileia in southwestern Greece, a seaside region located at a latitude of approximately 37.67 degrees north and an altitude of 13 to 50 m. The mean monthly duration of sunshine in this location varies from 143.2 h in January to 360.4 h in July [24]. Further north and south of the equator, the zenith angle of the sun is increased, resulting in a decrease in the amount of solar UV-B radiation reaching the earth. In our study, we found a high prevalence of vit D deficiency and insufficiency in the children, and we observed that increased age was associated with lower serum concentration of 25(OH)D. These results are in line with data from other research groups from sunny countries [25].

Although this study was conducted at a latitude of 37 degrees, during the winter months, the synthesis of 25(OH)D was lower than in summer, as reflected by the significantly lower mean values of serum 25(OH)D in winter and spring compared to summer and autumn. Our results are closer to the results from studies performed at 40 degrees of latitude than those at 33 degrees. One explanation could be the Western lifestyle in the study area, with a lot of indoor activities.

For a more accurate seasonal comparison, taking into account the local mean monthly hours of sunshine, we divided the months in 2 groups: low- and high-sunshine months [24]. Considering that the 25(OH)D status is a reflection of sun exposure during the preceding month, on the basis of the fact that the half-life of circulating 25(OH)D is 2–3 weeks [26], November to April were characterized as low-sunshine months and May to October were characterized as high-sunshine months. Significantly lower mean values of 25(OH)D were recorded in our population during the months November to April compared to the months May to October.

The mean 25(OH)D levels among the younger children, 1–4 years of age, were significantly higher than those in other age groups. The prevalence of a low concentration of 25(OH)D, corresponding to vit D deficiency and insufficiency, was 75% in the adolescents (13–18 years), with no gender difference. These findings are in line with other clinical reports. In adolescents from Kuwait, the prevalence of vit D deficiency was 81.21% and, there, it was significantly higher among girls compared with boys (91.69% vs. 70.32%; *p* < 0.001) [27].

In our study, exposure to sunlight during the months of November through April did not produce quantities of cholecalciferol in the skin enough to keep the level of 25(OH)D in the sufficiency range. These results are consistent with those of other studies, and comparable even with studies in higher latitudes, where the invoked cause of vit D insufficiency is sunshine quality. In our opinion, the cause for the low level of 25(OH)D recorded in the winter months and/or in low sunshine months, in our study, is related more to the duration of exposure to the sun, which is much lower than in the summer, and to the seasonal clothing style, which, during the winter, exposes only the face and hands. Turkish girls and premenopausal women wearing traditional clothing, with the skin of only the hands and face uncovered, were found to have lower vit D levels than those wearing lighter clothing, exposing the arms, but higher than those dressed in traditional Islamic style. These findings suggest that exposure to the sun of the skin of the hands and face may provide some vit D synthesis, but this may not be enough to eliminate vit D deficiency [28].

In order to protect against sunshine, almost all regulatory agencies recommend staying in the shade and wearing protective clothing, a hat with a wide brim, and sunglasses, as well as using sunscreens [29]. The implementation of sun exposure guidelines on shade and protective clothing was associated with lower vit D status among healthy adults, but not among children [30]. Empirically, we can say that the children playing on the beach are constantly coming out from the shade and not continuously wearing hats and protective clothing; consequently, these parameters in real life have an increased uncertainty for children. Similar to our results, Hansen et al. revealed that the use of sunscreens was not associated with low vit D levels, possibly due to more intense sun exposure [30].

Although no details were collected, we assumed that all the study children were subject to similar sunlight exposure in the preceding 12 months. In support of this hypothesis was the finding that, in the age groups 5–12 years and 13–18 years, the lowest levels of 25(OH)D were recorded in April and May (Figure 2). During these months, school activities are so intense that they allow children less time to play outside; specifically, for the age group 13–18 years, this is the period before the annual school assessment exams. In contrast, in May, the age group 1–4 years recorded the third highest mean value of 25(OH)D in the year. In August, the mean value was lower than in the adjacent months; one explanation for this could be the avoidance of exposure to sun, due to the extremely high temperatures in the middle of the day.

Promotion of sunscreen use is aimed at decreasing sunburn and preventing skin damage and skin cancer, although concerns have been raised regarding vit D synthesis. Some studies revealed that the use of sunscreen (SPF 8 and SPF 15) interfered with the cutaneous production of vit D3 [17], but others found no person who developed vit D levels outside the normal reference range after regular usage of sunscreens [31,32,33]. Kligman and colleagues reported that, in adults, self-reported sunscreen use was inversely associated with 25(OH)D3 levels [34]. A recent study of 2390 non-Hispanic white adults aged 20–59 years showed that sun-sensitive individuals more frequently used sun protection methods, including sunscreens, despite which their risk of vit D deficiency did not increase [35]. In healthy adults, sun exposure with sunscreen (SPF 30) caused similar vit D variation to that without photo protection [36]. Hence, consensus statements from various different countries have concluded that the results from studies based on real-life daily sunscreen application, which show no measurable reduction in vit D levels, outweigh the reports of diminished vit D synthesis observed under laboratory conditions [37].

One explanation for the finding of higher mean levels of 25(OH)D in children who used sunscreen both on and off the beach than in those who did not use creams, despite all children visiting the beach during the summer months, would be that the former were probably exposed to the sun for longer periods of time. It can be speculated that they have a lifestyle that includes more outdoor activities, in concordance with other publications. In a recent review, Passeron and colleagues concluded that sunscreen use for daily and recreational photoprotection does not compromise vit D synthesis, even when applied under optimal conditions [38]. Furthermore, sunscreens applied during a week-long holiday in a sunny country still allow a highly significant improvement of serum 25(OH)D3 concentration [39]. Even in less sunny countries, the use of sunscreen was associated with adequate vit D status [30,40] or was not shown to decrease it [41]. Although examined in adults, thus far, the correlation of vit D status in children and sun-protective measures in real-life conditions have not been well researched.

Depending on the time of day, season, latitude, and skin pigmentation, it has been shown that exposure of arms and legs for 5 to 30 min, between the hours of 10:00 a.m. and 3:00 p.m., twice a week, is adequate to provide a sufficient amount of vit D3 [6]. Some physicians recommend parents to allow their children 10 to 15 min of sun time before applying sunscreen on the beach, with the rationale that this gives them sufficient exposure to sunlight to meet their vit D requirements. Our data showed no difference in 25(OH)D levels between the group who applied sunscreen only on the beach and the group who also applied creams away from the beach. Reasons for these results could be that parents and children do not apply adequate amounts of sunscreen before going into the sun, or they forget to reapply sunscreen after several hours or after going into water. Infants and most children around the world usually wear light clothing during the summertime, not providing total skin cover at all times. Real-life sunscreen application, therefore, does not affect the level of vit D during the summer months, and parents should not be discouraged from the use of sunscreens for their children [41]. No child in our study had application of sunscreens during the low-sunshine months (November–April), when they usually did not go to the beach and they wore skin-covering clothes, both factors that influence vit D production. During these months, their levels of 25(OH)D were lower than during the high-sunshine months (May to October) when they used sunscreens. Perhaps, there is a need to look for causes of low levels of 25(OH)D other than use of sunscreens, such as the time spent indoors during the sunshine hours. We can conclude from the findings of this study that sunscreen use does not interfere with production of sufficient vit D in children in this part of Greece in the high-sunshine months, and that vit D supplementation should be encouraged during the low-sunshine months. The European Academy of Pediatrics (EAP) recommends, for prevention in infants (0–1 year) and children (1–18 years) at risk of vitamin D deficiency, 400–1000 IU/day and 600–1000 IU/day, respectively [42].

### Strengths and Limitations of the Study

The main strength of the paper is that it presents the first real-life study performed in the southern region of Greece investigating the effect of sunscreen application on vit D level. One limitation of this study was the small sample size of children, and that sequential measurements were not made for each child.

We were not able to investigate the possible influence on 25(OH)D levels of time spent outdoors and indoors, of duration of exposure to the sun, or of the combination of shade, clothing, and sunscreen. The answers regarding these parameters in real life for children living in seaside regions have increased uncertainty, as mothers could answer if the children had a protective clothing, hat, or sunglasses but they could not specify how long they really used them. The study did not evaluate how frequent the sunscreen was applied on the beach or its amount. In addition, the authors did not assess the dietary habits which can influence the 25(OH)D level.

## 5. Conclusions

In children, the prevalence of vit D deficiency and insufficiency in a sunny region is high and shows seasonal variation, with normal serum levels of 25(OH)D during the high sunshine months and low levels in the low sunshine months. Our data suggest that the use of sunscreen during the summer allows sufficient sunlight to be received by the skin to enable the production of vit D at a level capable of maintaining serum levels of 25(OH)D within normal limits in the months of sunshine. Thus, in real life, the use of sunscreens during the summer months is not a risk factor for vit D deficiency. Parents and children should be encouraged to use sunscreens when they are exposed to sunshine. Vit D supplements should be given to the children during the low-sunshine months.

## Figures and Tables

**Table 1:** Demographic data and vitamin D level according to patient gender, season, and use of sunscreen.

Parameter	*N* (%)	VitD Mean (SD)	*p*
Gender			
Males	184 (48.9)	27.5 (10.6)	0.156
Females	192 (51.1)	26 (10.1)	
Month			
January	31 (8.2)	17.9 (6.8)	*p* < 0.001 ^++^
February	24 (6.4)	21.8 (7.4)	
March	29 (7.7)	22.7 (5.8)	
April	24 (6.4)	20.6 (7.5)	
May	35 (9.3)	26.5 (13.9)	
June	39 (10.4)	27.6 (10.4)	
July	40 (10.6)	33 (9.4)	
August	25 (6.6)	30.1 (8.2)	
September	44 (11.7)	34.6 (8.7)	
October	28 (7.4)	30.1 (10.6)	
November	38 (10.1)	25.7 (6.9)	
December	19 (5.1)	20.5 (9.4)	
Season			
Winter	74 (19.7)	19.8 (7.8)	*p* < 0.001 ^++^
Spring	88 (23.4)	23.6 (10.4)	
Summer	104 (27.6)	30.3 (9.7)	
Autumn	110 (29.3)	30.4 (9.4)	
Sunscreen ex beach			
Yes	76 (20.2)	24.9 (10)	*p* < 0.001 ^+^
No	300 (79.8)	33.8 (8.6)	
Sunscreen on the beach			
Yes	128 (34.05)	23.7 (9.8)	*p* < 0.001 ^+^
No	248 (65.95)	32.5 (8.9)	

^+^ Student’s *t*-test; ^++^ ANOVA.

**Table 2:** Vitamin D (vit D, 25(OH)D) deficiency and insufficiency in healthy children in Greece, according to demographic characteristics, month, and season (*n* = 376).

	Vitamin D levels			
	Deficiency *N* (%)	Insufficiency *N* (%)	Normal *N* (%)	*p*
Gender				
Males	46 (25)	70 (38)	68 (37)	0.410 ^+^
Females	56 (29.2)	77 (40.1)	59 (30.7)	
Age, Mean (SD)	9.1 (4.7)	7.6 (4.9)	6.4 (4.9)	*p* < 0.001 ^++^
Month				
January	20 (64.5)	9 (29)	2 (6.5)	*p* < 0.001 ^+^
February	11 (45.8)	10 (41.7)	3 (12.5)	
March	8 (27.6)	17 (58.6)	4 (13.8)	
April	13 (54.2)	7 (29.2)	4 (16.6)	
May	12 (34.3)	13 (37.1)	10 (28.6)	
June	6 (15.4)	23 (59)	10 (25.6)	
July	3 (7.5)	13 (32.5)	24 (60)	
August	1 (4)	13 (52)	11 (44)	
September	1 (2.3)	13 (29.5)	30 (68.2)	
October	5 (17.9)	9 (32.1)	14 (50)	
November	10 (26.3)	17 (44.8)	11 (28.9)	
December	12 (63.2)	3 (15.7)	4 (21.1)	
Season				
Winter	43 (58.1)	22 (29.7)	9 (12.2)	*p* < 0.001 ^+^
Spring	33 (37.5)	37 (42)	18 (20.5)	
Summer	10 (9.6)	49 (47.1)	45 (43.3)	
Autumn	16 (14.5)	39 (35.5)	55 (50)	
Sunscreen ex beach				
Yes	2 (2.6)	25 (32.9)	78 (26)	*p* < 0.001 ^+^
No	100 (33.3)	122 (40.7)	33.8 (8.6)	
Sunscreen on the beach				
Yes	6 (4.7)	49 (38.3)	73 (57)	*p* < 0.001 ^+^
No	96 (38.7)	98 (39.5)	54 (21.8)	

^+^ Pearson’s chi-square test; ^++^ ANOVA.

## Data Availability

Data available on request due to restrictions (privacy and ethical restrictions).
